# A Retrospective Operative and Early Outcome Comparison of Suprapubic Transvesical Prostatectomy and Transurethral resection of the Prostate

**DOI:** 10.4314/ejhs.v31i4.13

**Published:** 2021-07

**Authors:** Andualem Beyene, Abeselom Lemma, Seyfe Tilahun

**Affiliations:** 1 Department off Surgery, School of Medicine, College of Health Sciences, Addis Ababa University

**Keywords:** Suprapubic transvesical prostatectomy, Transurethral resection of prostate, Operative and early outcome

## Abstract

**Background:**

BPH is the major cause of bladder outlet obstruction over the age of 40 years. Multiple surgical management options have been described of which STVP and TURP are the oldest and widely available procedures. The objective of this study is to describe and compare the intraoperative and early outcome situations of STVP and TURP.

**Methods:**

This is a hospital-based retrospective descriptive study that compares intraoperative and early outcomes of STVP and TURP in Menilik II Hospital from January 2017 to December 2019. The study samples were 72 STVP and 72 TURP patients.

**Results:**

The mean duration of surgery in the STVP group was 97.8 minutes which is significantly longer than TURP group (66.15 minutes). Duration of post-op catheterization and hospital stay are significantly longer in STVP than TURP.

**Conclusion:**

The duration of surgery, length of hospital stays and post op catheterization are longer in STVP. There was no significant difference in intra-op and early complications from STVP and TURP.

## Introduction

Benign prostatic hyperplasia (BPH) is one of the common conditions in men seen after the age of 40 years and the incidence increases with age. It is a major cause of lower urinary tract symptoms (LUTS) and bladder outlet obstruction (BOO) (1).

Indications for surgical intervention include bothersome symptoms, failed medical treatment, recurrent urinary tract infections, recurrent hematuria, acute urinary retention, bladder stones, bladder diverticula, and upper urinary tract dysfunction. Several treatment options ranging from conservative treatment, medical treatment, minimally invasive, and invasive (open) surgical treatments have been described for patients with BPH (1).

Open prostatectomy and transurethral resection of the prostate (TURP) are time tested options of surgical management for BPH. Bruskewitz R et al. described that in the USA open prostatectomy accounted only for 3% of interventions for symptomatic BPH(2). Though TURP is considered the reference standard surgical treatment for BPH, open prostatectomy is still the mainstay of surgical management in many developing countries, including Ethiopia (3).

Open prostatectomy is the oldest surgical treatment for BPH that involves enucleation of prostate adenoma through open suprapubic transvesical or retropubic approach. capsule (3,4). TURP was first described in 1932 and involves endoscopic resection of prostate adenoma. (5). It is currently taken as the gold standard treatment for BPH(6).

The choice of surgical procedures for relief of BOO secondary to BPH depends on several factors including the availability of expertise and instruments, morbidity of the procedure, cost of the procedure, and the need for hospitalization and anesthesia (3).

In Ethiopia, TURP has been introduced recently.ers. Currently the choice between invasive and minimally invasive options of management partly depends on outcomes and complications from the available procedures. So far, there are no studies in Ethiopia that compared the two main surgical procedures; suprapubic transvesical prostatectomy (STVP) and TURP. In this study we aimed at comparing perioperative and early complications from TURP and STVP done for patients with BPH. The objective of the study isto describe intraoperative and early outcomes of STVP and TURP. In addition, the study tries to compare the intraoperative conditions the early outcomes during STVP and TURP.

## Methods

This is a retrospective study that compared intraoperative and early outcomes of STVP and TURP in Minelik II hospital from January 2017 G.C. to December 2019 G.C. at Menilik II Hospital, a referral Hospital in Addis Ababa, Ethiopia. The source population is all patients who underwent STVP and TURP from January 2017 G.C. to December 2019 G.C.

In the study period a total of 194 patients underwent surgery for BPH of which 110 are TURP and 84 STVP. A sample of 77 patients from each group was enrolled in the study. Patients who underwent STVP or TURP for non-BPH condition, and patients who have history of previous urethral or prostate surgery were excluded from the study. Data was collected from patient records on a predesigned questionnaire and entered in SSPS version 21 for analysis.

The study was with ethical approval from the department of Surgery research and publication committee. All patient identifiers like name were not included. The limitation of the study is that it is a single hospital based retrospective study.

## Results

The mean age of presentation was 63.53 and 66.76 years in TURP and STVP groups respectively with no statistically significant difference.

Poor urinary stream, frequency, nocturia, and dribbling were the commonest presenting LUTS in both groups, accounting for 95.8%, 79.2%, 75.0%, 70.8% in TURP group and 93.1%, 80.6%, 76.4% and 61.1% in STVP group respectively. In the STVP group, 62.5% of the patients had pre-operative acute urinary retention (AUR) which was significantly higher than the TURP group (26.4%). Hypertension and Diabetes Mellitus were the commonest co-morbidities in both groups accounting for 27.8% and 8.3% in the TURP group, and 16.7% and 9.7% in the STVP group (Table 1).

**Table 1:** Clinical presentation of patients in TURP and STVP groups

Variable	TURP (n=72)	STVP (n=72)	P-value
Mean age (years)	63.53	66.76	.059
Pre-op symptoms			
Poor stream	69(95.8%)	67 (93.1%)	0.467
Straining	18(25.0%)	16(22.2%)	0.695
Hesitancy	31(43.1%)	32(44.4%)	0.867
Dribbling	51(70.8%)	44(61.1%)	0.218
Acute retention	19(26.4%)	45(62.5%)	0.000
Chronic retention	5(6.9%)	1(1.4%)	0.095
Frequency	57(79.2%)	58(80.6%)	0.835
Nocturia	54(75.0%)	55(76.4%)	0.846
Urgency	40(55.6%)	42(58.3%)	0.736
Hematuria (gross)	5(6.9%)	2(2.8%)	0.245
Urge incontinence	6(8.3%)	9(12.5%)	0.413
Overflow incontinence	1(1.4%)	0	0.316
Co-morbidity			
Hypertension	20(27.8%)	12(16.7%)	0.109
Diabetes mellitus	6(8.3%)	7(9.7%)	0.771
Asthma	1 (1.4%)	3(4.2%)	0.310
Cardiac illness	2(2.8%)	4(5.6%)	0.404

The mean prostatic volume was 93.78 ml and 50.27 ml in the STVP and TURP groups, respectively. Pre-operatively 39 patients (54.2%) in the STVP group and 24 patients (33.3%) in TURP group had history of catheterization for urinary retention. The mean duration of catheterization was 45.96 days and 34.44 days in TURP and STVP groups respectively (Table 2).

**Table 2:** Pre-operative factors in both groups

Variable	TURP n=72	STVP n=72	P-value
Prostate volume (ml)	50.27	93.78	0.000
Pre-op catheterization	24 (33.3%)	39 (54.2%)	0.012
Mean duration of catheterization (days)	45.96	34.44	0.215
Mean pre –op Hemoglobin (g/dl)	15.24	14.4	0.028
Renal impairment (Seum Cr>1.5)	6 (8.3%)	0	0.012
Hydronephrosis	6(8.3%)	3(4.2%)	0.302

Out of the 72 patients in TURP group, 6 (8.3%) had serum creatinine level of greater than 1.5 while none STVP group. Six patients (8.3%) in the TURP group and 3 patients (4.2%) in the STVP group had hydronephrosis in pre-operative ultrasound imaging (Table 2).

**Figure 1:**
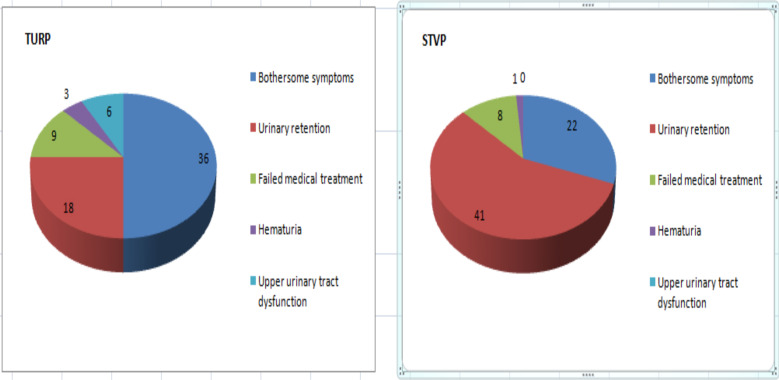
Indications for surgery in TURP and STVP groups

The most common indications for surgery were urinary retention followed by bothersome symptoms in the STVP group while bothersome symptoms followed by urinary retention were the most common indications in the TURP group (Figure 1).

**Figure 2:**
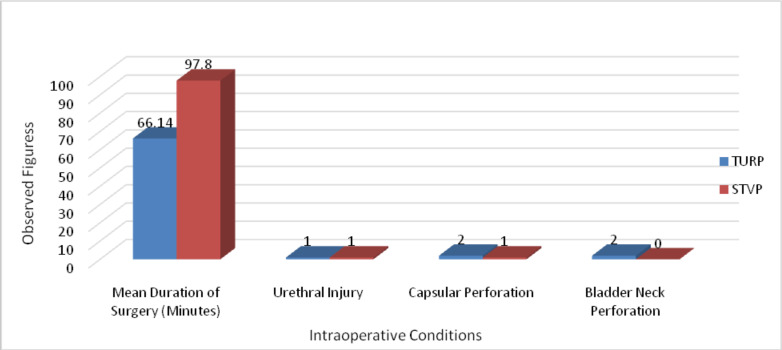
Intra-operative conditions in TURP and STVP groups

The mean duration of surgery in the STVP group was 97.8 minutes which is significantly longer than in TURP group (66.15 minutes). Intra-operatively one patient each from the TURP and STVP groups had urethral injury. Capsular perforation occurred in two patients in TURP group and in 1 patient in STVP group. Two patients in the TURP group had bladder neck perforation recognized intra-operatively (see Figure 2).

The mean duration of post-op catheterization is 12.2 days and 3.4 days in the STVP and TURP groups, respectively. The mean duration of both post-operative and total hospital stay was significantly longer in the STVP group than the TURP group (Table 3).

**Table 3:** Post-operative conditions and complications in TURP and STVP groups

Variable	TURP (n=72)	STVP(n=72)	P-value
Mean duration of post-op catheterization (days)	3.37	12.18	.000
Mean duration of post-op hospital stay (days)	2.94	4.08	.000
Mean duration of total hospital stay (days)	6.67	8.02	.012
Blood transfusion	1 (1.4%)	1 (1.4%)	1.000
Clot retention	1 (1.4%)	0	.316
UTI	1 (1.4)	5 (6.9%)	.095
Urgency	21 (29.2%)	21 (29.2%)	1.000
Urinary retention after catheter removal	2 (2.8%)	2 (2.8%)	1.000
Urinary incontinence	2 (2.8%)	13 (18.1%)	.003
Urge incontinence	2 (2.8%)	9 (12.5%)	.028
Stress incontinence	0	3 (4.2%)	.080
Total incontinence	0	1 (1.4%)	.316

One patient from each group needed blood transfusion post-operatively. One patient from the TURP group developed clot retention post-operatively. Post-operatively urinary tract infection occurred in 5 patients in STVP group and in 1 patient in the TURP group. The most common lower urinary tract symptom in the early post-op period was urgency occurring in 29.2% of patients in both groups. One patient developed suprapubic urinary leakage which was managed with prolonged catheterization (Table 3).

After catheter removal two patients from the TURP and STVP groups each developed urinary retention. The post-operative incidence of urge urinary incontinence was higher in STVP group (12.5%) than in the TURP group (2.8%) (Table 3).

## Discussion

The usual patients operated for benign prostatic hyperplasia are elderly. In our study, the mean age of presentation is 63.53 and 66.76 years in TURP and STVP groups respectively with no significant difference between the two groups. However other RCT reported significant age differences in the two groups (8,9).

Hypertension and diabetes mellitus were found to be the most common co-morbid illnesses in the TURP group, and the STVP group respectively. Similarly hypertension and diabetes mellitus were the commonest co-morbidities in a study from Kenya with incidence of 29% and 13% respectively (10).

The indications for surgery in benign prostatic hyperplasia were similar with other studies in Ethiopia (7, 12). In other studies in addition to the indication we found failure of medical management is a common indication for surgery (8,11).

The mean prostatic volume was different from other studies. A higher prostate volume is reported from a randomized study in china with average prostate volume of 131.0 ml and 138.4 ml in TURP and STVP groups respectively (9). The mean duration of surgery in this study has a significant difference suggesting a longer operating time in STVP. Studies differ in the duration of surgery for both groups (9, 14, 15).

The study found that the mean duration (in days) of both post-operative and total hospital stay was significantly longer in the STVP group than the TURP group). Similar findings are reported from other studies (7,9,10). The mean duration of post-op catheterization is in the STVP group is significantly longer than in the TURP group. Similarly, in other studies, the duration of post-op catheterization is reported to be longer in patients who underwent STVP (9,10,15,16).

Bleeding can occur both intraoperatively and postoperatively. The amount of blood loss may depend on gland size and resection weight(17). In our study, only 2 patients (1 from each group) needed blood transfusion which is a lower rate compared to the available literature (19,20,21). Another study from Ethiopia found a 4.6% rate of transfusion after STVP(7). A similar finding is reported by Sagarkumar Gupta et al with no significant difference in rates of transfusion with a rate of 8% and 12% for STVP and TURP respectively (22)

Capsular perforation as a complication is within the range of other studies. In this study, clot retention was low in both groups and other studies showed different rates of clot retention. (7, 12, 23). A similar study of complications of STVP in Nigeria by Oranusi et al showed that 5 out of 362 patients (1.4%) developed clot retention which was managed by re-exploration (24). In their RCT, Sagarkumar Gupta et all reported clot retention rates of 12% and 8% for STVP and TURP respectively with no significant difference (22). Other studies reported a higher rate of clot retention in TURP than for STVP (8, 15).

The rate of UTI in the early post-op period was almost similar with other studies in other parts of the world but Sagarkumar Gupta et al reported higher rates of UTI in TURP (8, 9, 21, 22, 25).

A leak from suprapubic site after STVP is rare and a similar finding is reported by in a descriptive study of TVP(12). The reported rates of suprapubic leak after STVP in other literatures is range from 0.4% to 16% (22,26,27).

The rate of wound infection after STVP in this study is low but it varies in other studies which reported a wound infection rate ranging from 1.2% to 34% (2,20,28). Failure to void after catheter removal is similar with finding in other studies (23, 28). However, there are reports with a urinary retention rate ranging from o to 34% (18,29,30).

Urinary urgency is similar for both TURP and STVP groups. The rate of urge incontinence after STVP was significantly higher in the STVP group than TURP group. A relatively higher rate is reported in a randomized trial, 53% of STVP patients and 49% TURP patients (16). Lower rates of incontinence are reported from a randomized study in Iran and China (8,9).

In conclusion, in this study we found that the duration of surgery, length of hospital stay, length of post-op catheterization was higher in patients who underwent STVP than in those patients who underwent TURP. There was no significant difference in the incidence of intra-operative and early post-operative complications except for post-op urge incontinence which is higher in the STVP group. From this study, we recommend that in resource limited setups, suprapubic transvesical prostatectomy can safely be offered for patients with BPH.
